# Strengthening of Reinforced Concrete Beams with Externally Mounted Sequentially Activated Iron-Based Shape Memory Alloys

**DOI:** 10.3390/ma12030345

**Published:** 2019-01-22

**Authors:** Emanuel Strieder, Christoph Aigner, Gabriele Petautschnig, Sebastian Horn, Marco Marcon, Michael Schwenn, Oliver Zeman, Pablo Castillo, Roman Wan-Wendner, Konrad Bergmeister

**Affiliations:** 1 Institute of Structural Engineering, Department of Civil Engineering and Natural Hazards, University of Natural Resources and Life Science (BOKU), Vienna 1190, Austria; christoph.aigner@students.boku.ac.at (C.A.); gabriele.petautschnig@students.boku.ac.at (G.P.); marco.marcon@boku.ac.at (M.M.); michael.schwenn@boku.ac.at (M.S.); oliver.zeman@boku.ac.at (O.Z.); pablo.castillo@boku.ac.at (P.C.); roman.wendner@boku.ac.at (R.W.-W.); konrad.bergmeister@boku.ac.at (K.B.); 2 IPEK—Institute of Product Engineering at Karlsruhe Institute of Technology (KIT), 76131 Karlsruhe, Germany; sebastian.horn@partner.kit.edu; 3 Department of Structural Engineering, Ghent University, 9052 Ghent, Belgium

**Keywords:** iron-based shape memory alloys, strengthening RC beams, sequential activation, infrared heating

## Abstract

Iron based shape memory alloys (Fe-SMA) have recently been used as active flexural strengthening material for reinforced concrete (RC) beams. Fe-SMAs are characterized by a shape memory effect (SME) which allows the recovery of previously induced plastic deformations through heating. If these deformations are restrained a recovery stress is generated by the SME. This recovery stress can be used to prestress a SMA applied as a strengthening material. This paper investigates the performance and the load deformation behavior of RC beams strengthened with mechanical end anchored unbonded Fe-SMA strips activated by sequentially infrared heating. The performance of a single loop loaded and a double loop loaded SMA strengthened RC beam are compared to an un-strengthened beam and a reference beam strengthened with commercially available structural steel. In these tests the SMA strengthened beam had the highest cracking load and the highest ultimate load. It is shown that the serviceability behavior of a concrete beam can be improved by a second thermal activation. The sequential heating procedure causes different temperature and stress states during activation along the SMA strip that have not been researched previously. The possible effect of this different temperature and stress states on metal lattice phase transformation is modeled and discussed. Moreover the role of the martensitic transformation during the cooling process on leveling the inhomogeneity of phase state in the overheated section is pointed out.

## 1. Introduction

Shape memory alloy (SMA) strips can be used as prestressing elements to strengthen reinforced concrete (RC) members. The shape memory effect (SME) turns a material that is deformed beyond the elastic range back into its initial shape [1,2,3]. If reshaping is prevented by mechanical fixation a stress develops within the shape memory alloy, which is called recovery stress. The application of SMAs as prestressing elements is still in an early research state [4,5]. Iron based SMAs have been used for near surface mounted strengthening of concrete beams [6] and embedded strengthening [7]. One possible solution of flexural strengthening with end anchored SMA strips is discussed in [8,9]. SMA strips have also been tested as strengthening systems for steel plates [10]. The creep and relaxation behavior of this kind of alloys under low temperatures is investigated in [11].

Under research conditions the activation of the SMA is often done simultaneously through the whole SMA by applying electric current [5,6,10]. For a constant cross-section the electrical resistance causes a homogeneous heating process within the whole SMA. This is essential as the phase change in the SMA requires a certain temperature level. For long SMA members with a wide cross-section the resistive heating method is limited because the required voltage increases linearly with the heated length and the cross section affects the current [5]. For practical use the activation procedure should be easy to apply at a job site, it should not be dangerous in use (high currents and voltage) and it should be economical. An alternative is the local activation by infrared (IR) light.

The effects of sequential heating of the SMA in strengthening application have not been researched so far. Sequential heating generates widely varying temperatures during the heating and cooling processes. Due to the fact that the phase transformation of the SMA is temperature and stress dependent, the sequential activation causes a more complex transformational behavior of the SMA than a homogeneous activation procedure.

**Figure 1:**
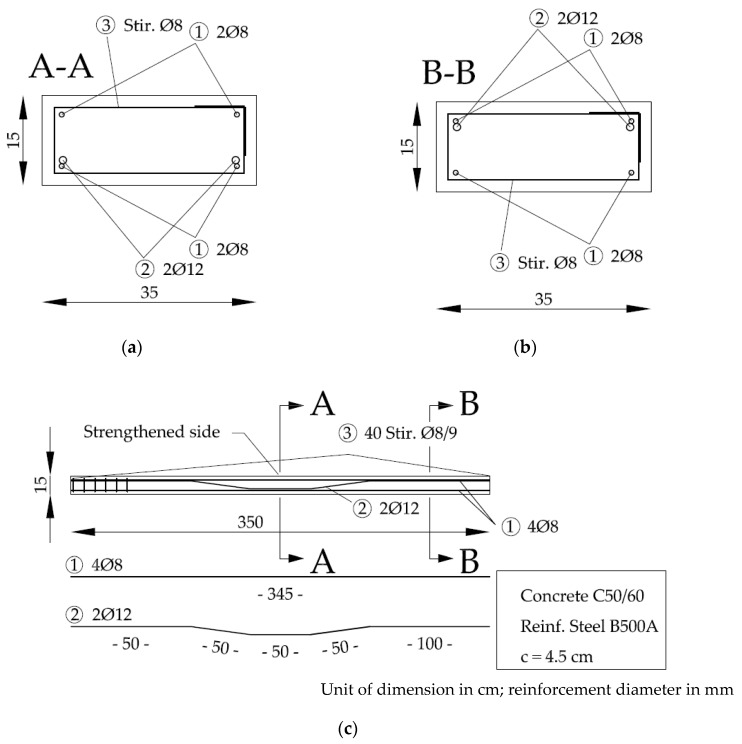
(**a**) Cross section of the reinforced concrete beam in the strengthened area; (**b**) Cross section of the not strengthened area of the reinforced concrete beam; (**c**) Longitudinal cut of the reinforced concrete beam.

In this study two reinforced concrete beams strengthened with prestressed, unbonded SMAs are investigated. The material used is an iron-based SMA combined with infrared heating as activation technique. Two reference RC beams, one without additional external strengthening at all (only mild reinforcement as depicted in Figure 1) and one externally strengthened with structural steel with a yield point of about 500 MPa, were tested. The main outputs are (i) the analysis of a potential performance increase associated with externally strengthening a reinforced concrete beam with iron-based SMA strips that are pre-stressed by thermal activation using infrared heating; (ii) the investigation of the effect of sequential activation on prestressing and phase transformation of the SMA used as a prestressing material.

## 2. Materials and Methods

### 2.1. Materials

#### 2.1.1. Concrete

The reinforced concrete specimens used in four point bending tests were beams of the strength class C50/60. The geometry of the reinforced concrete beam is shown in Figure 1 and the concrete composition for the C50/60 is given in Table 1. The classification of the used aggregates according to EN 932-3 is given in Table 2.

The material parameters of the concrete were determined as follows. The concrete compressive strength was tested according to EN 12390-3 [12] and the tensile strength according to EN 12390-6 [13] on cylinders with a height of 150 mm and a diameter of 100 mm. The fracture energy was tested according to ÖNORM B 3592 [14] on cubes with a side length of 150 mm by means of a wedge splitting test. The mean value results (calculated from three specimens) of the concrete compressive strength, tensile strength, and fracture energy are listed in Table 3.

The reinforcement position 2 in Figure 1 is used to avoid bending cracks in the cross sections without strengthening and is anchored into the concrete compression zone in the strengthened area.

#### 2.1.2. Shape Memory Alloy for Strengthening

The SMA used in the tests was an iron-based shape memory alloy containing manganese (Mn) silicon (Si) and other constituents. The alloy composition and the exact material properties of the used material batch is not part of this research since it focuses more on the application of the alloy, even though it is very important to consider the material behavior for a strengthening application. A detailed material characterization of shape memory alloys for use in strengthening application is carried out in [15,16]. The phase transformation temperatures can be tested as stated in [17]. Fatigue behavior of SMA was studied in [18] and the fatigue of SMA strengthened concrete beams was researched in [19]. The properties of the alloy used in this study may vary from the composition in other research sources.

**Figure 2:**
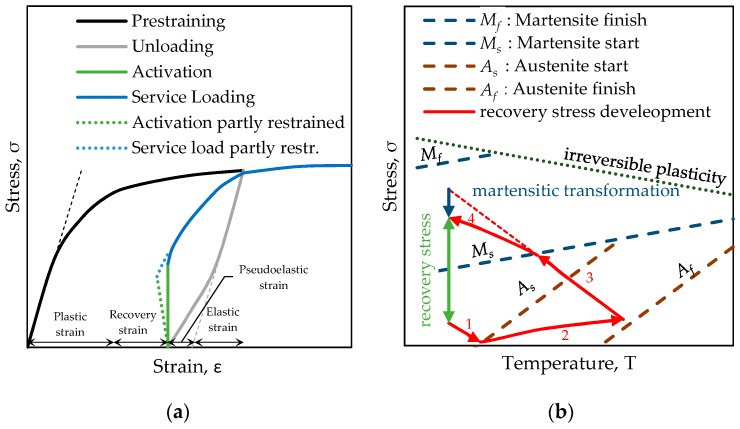
(**a**) Schematic sketch of the stress-strain response during shape memory alloy (SMA) application for prestressing, adapted from [15]; (**b**) schematic illustration of the activation and recovery stress development, adapted from [15].

The interesting behavior of this type of iron based SMA is the shape memory effect (SME) and the potentially generated recovery stress, respectively. If a SMA is stretched uniaxially beyond the linear elastic region (black curve in Figure 2a) and unloaded again (grey curve in Figure 2a) a certain strain remains. In common metal alloys the remaining strain is due to plastic deformation. In the iron based SMA the metal lattice structure is transformed from austenite to martensite in this deformation process. If the unloaded material is heated to a certain transformation temperature a reverse phase transformation takes place. After heating and cooling down, the material recovers the deformation that is associated with the phase transition, the recovery strain. This phenomenon is called shape memory effect. If the deformation of the SMA strip is restrained, the SME generates a recovery stress (green line in Figure 2a). The blue line in Figure 2a represents the behavior of the material due to further loading.

The process of thermal activation of a restrained SMA is displayed in Figure 2b (temperature on the abscissa and stress on the ordinate). The initial stress level in the scheme is required to avoid compression and buckling of a tensile component. The recovery stress development starts with a stress decrease due to thermal expansion until the austenite start temperature (*A_s_*) is reached (part 1 of the red line in Figure 2b). Further heating between *A_s_* and austenite finish temperature *A_f_* leads to an increase in stress due to the SME (part 2 of the red line in Figure 2b). During the cooling process an almost linear stress increase occurs due to thermal contraction of the metal until the martensite start temperature (*M_s_*) criterion is met (part 3 of the red line in Figure 2b). This behavior is the reverse effect of thermal expansion observed in common metals. A further cooling of the SMA still increases the recovery stress but is dampened by the martensitic transformation (part 4 of the red line in Figure 2b). The recovery stress is defined as the change in stress before and after activation (green line in Figure 2b). In reality the SMA is not perfectly restrained: the SME is converted, depending on the stiffness of the RC member and the clamping devices into recovery strain (reshaping) and recovery stress (prestressing) as can be seen from the green dotted line in Figure 2a. This phenomenon is also explained in [10].

#### 2.1.3. Strengthening Steel

One RC beam that was otherwise identical to the beams tested with SMA served as reference beam. It was strengthened with a commercially available structural steel strip with a yield strength of 500 MPa. The geometry of the steel strip was identical with the one of the SMA strip.

### 2.2. Test Setup Measurements and Test Procedure

#### 2.2.1. Preparation of the SMA Strips

The SMA strip used in the tests had a nominal thickness of 2.3 mm, was nominally 50 mm wide and 1500 mm long before pre-straining. The ends of the strip were shaped as shoulders in the style of a tensile specimen (see Figure 14b). In order to prepare the SMA for later application as pre-stressing element the phase transformation needs to be triggered by pre-straining the strip. In this investigation all SMA strips were pre-strained in force-control using a hydraulic jack. The force was measured by a load cell and a linear variable differential transformer (LVDT) controlled the displacement.

**Figure 3:**
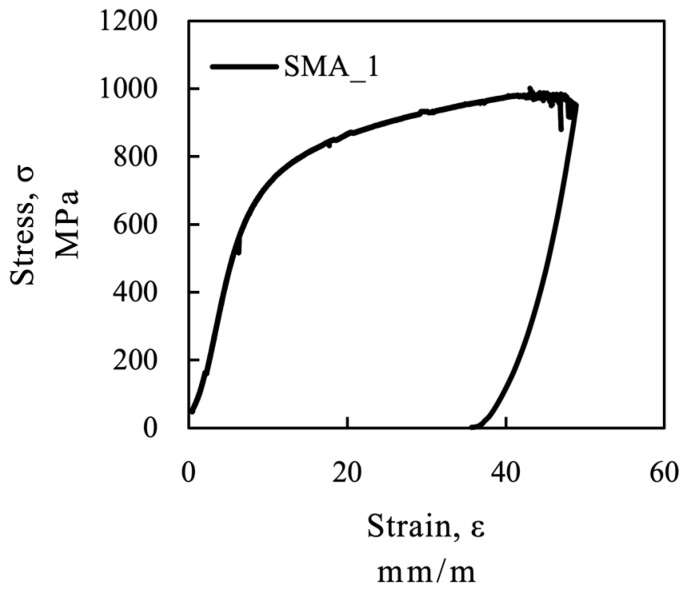
SMA during prestraining.

The residual strain after pre-straining and unloading of the SMA samples is shown in Table 4 and a diagram of the SMA during pre-straining is presented in Figure 3. The stress is calculated based on the initial cross section of the strip *A_0_* and the strain is assumed to be constant over the length of the strip.

#### 2.2.2. Anchoring of the Strengthening Strip to the Concrete Beam

**Figure 4:**
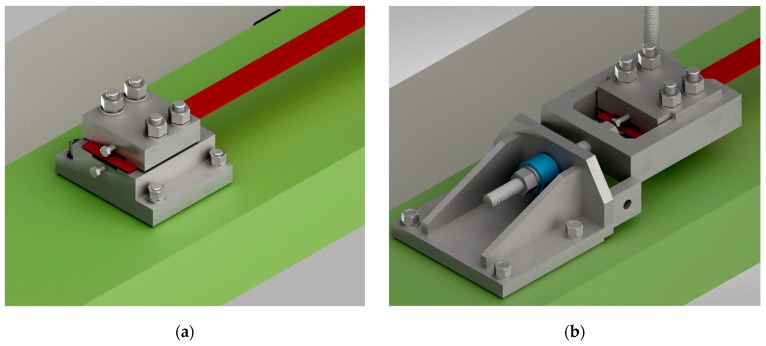
(**a**) SMA strip (red), fix clamping and anchorage device (grey) fixed to the concrete (green), (**b**) clamping and anchorage device (grey) with load cell (blue) to measure the force in the SMA strip.

A special clamping device was developed for this study. The clamping and anchoring device was designed to transfer the force from the strengthening strip to the concrete member and allow the direct measurement of the obtained prestressing force using a load cell. Further design requirements for the clamping and anchoring device were (i) high stiffness to minimize loss of recovery stress due to deformations of the clamping and anchoring device, and (ii) adjustability in order to compensate for imperfections in the length of the strip and the position of the installed clamping device. The geometry of the clamping device is shown in Figure 4.

The clamping device was fixed to the concrete by use of bonded anchors. The annular gap between the clamping device and the thread of the bonded anchor was filled with mortar, to avoid an initial shift between the clamping device and the anchor.

#### 2.2.3. Test Setup

The concrete beams were tested in a four point bending test setup. Two of the beams were strengthened with SMA before testing, one was tested with a structural steel (yield point about 500 MPa) and one was tested without any strengthening. The test procedure is summarized in Table 5.

**Figure 5:**
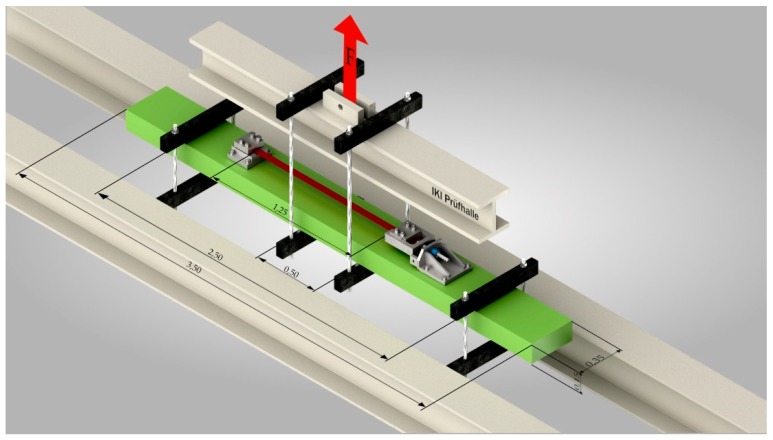
Test setup in four point bending test, dimensions in meters.

A sketch of the test setup is shown in Figure 5. As can be seen in the figure, the force was applied by one single cylinder on the top of the test setup. The upward bending of the beam was chosen to have better accessibility to the SMA strip for thermal activation and non-contact measurements during activation and structural testing.

#### 2.2.4. Testing Procedure

The strengthening strips were used as external tensile strengthening of a concrete beam in bending. For the test setup the SMA strip was clamped at its shoulders by the clamping device which is fixed to the concrete.

The concrete beam was pre-loaded to 1 kN before the activation of the SMA. This creates a force of approximately 5 kN in the SMA strip which defines a stress level of about 40 MPa for the nominal cross section of the SMA. This was done to avoid compressive stresses and buckling due to the initial thermal expansion of the SMA before the phase transformation from martensite to austenite, see also Figure 2b.

**Figure 6:**
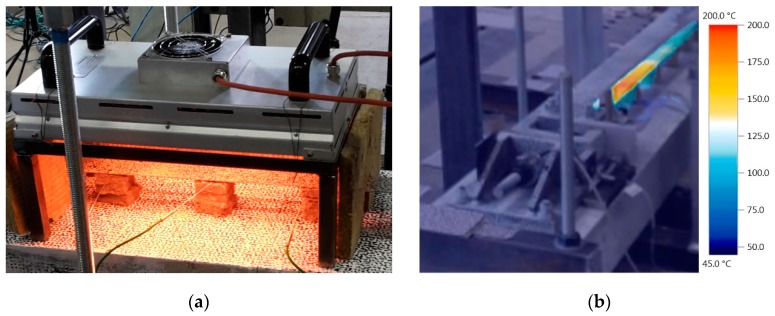
(**a**) Infrared emitter used as heating device for activation of SMA; (**b**) temperature monitoring using infrared camera (picture without correction of emissivity).

Resistive heating by applying electrical power to the material and utilizing the electrical resistivity of the material is commonly known [5,6,10]. Also heating by a oxy-acetylene rosebud has been used before [19,20]. In this study an infrared light emitter as heating device is employed and actives the alloy. A commercially available heater type “Heraeus MMS C-V 3500” with a length of 52 cm, a width of 25 cm, and a heating power of 3500 W was used. The distance from the heater to the SMA strip was 7 cm and the distance from the alloy to the concrete was 8 cm. The distance between heater and alloy influences the heating time. A higher distance leads to a longer heating procedure and a higher relevance of heat transfer within the metal. Thermal insulation on three sides ensured minimum thermal losses and disturbing light influence on the digital image correlation measurements, see Figure 6a.

The heating procedure was done sequentially due to the fact that the SMA strip was longer than the heater. Once the target temperature was reached at one thermocouple the infrared emitter was moved to the next heating position. Figure 8 shows these heating and cooling measurements. After the heating procedure, the strip was cooled down to the ambient temperature. During the whole heating and cooling procedure the displacement at the supports and the loading points was restricted. The deformation was recorded due to the fact that the beam can deform between the four fixed points. After the SMA strip was cooled to ambient temperature, the strengthened and now prestressed beam was loaded displacement controlled in the four point bending test setup. The beam “SMA_1” was loaded to ultimate load in this test step. The beam “SMA_2” was loaded to 30 kN and then afterwards unloaded. A second activation of the SMA strip was performed. After the cooling of the SMA, the beam was loaded to ultimate load. This second activation cycle demonstrates the rehabilitation behavior of a damaged beam, strengthened with Fe-SMA.

#### 2.2.5. Measurements and Monitoring

**Figure 7:**
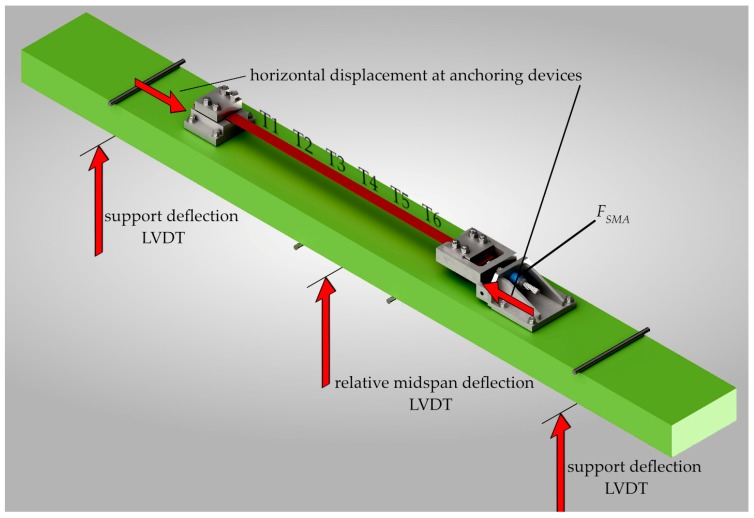
Monitoring setup at the beam. LVDT: linear variable differential transformer; *F_SMA_*: force in the SMA strip.

During the heating and cooling process the temperature in the SMA strip was measured using type K thermocouples. The position of the thermocouples (T1 to T6) can be seen in Figure 7. The distance between the 6 thermocouples was roughly 18 cm. Since the thermocouples are measuring temperatures in a small spot only, an infrared camera was used to monitor the temperature on a wider range, see Figure 6b. The infrared camera cannot be used for proper monitoring during the heating process due to the infrared emitting heater.

The vertical displacement at the supports and in the center of the beam was measured with a linear variable differential transformer (LVDT) to calculate the vertical deflection of the beam considering the stiffness of the test setup. Another two LVDTs were placed at the top of the concrete beam next to the anchoring devices to monitor the relative horizontal shift of the devices due to the force in the SMA strip.

The force in the SMA strip (*F_SMA_*) was measured with a 200 kN load cell. The vertical force (*F*) applied to the RC beam during the four point bending test was measured with a load cell with a measurement range of 630 kN. The deformation state of the SMA strip and the concrete beam during activation of the SMA and the four point bending test was monitored by three 3D digital image correlation systems consisting of high resolution cameras with defined view angles. The first system captured the front and top of the overall test setup, the second a detail of the SMA strip and the third was monitoring the clamping and anchoring device. This type of deformation measurement was chosen, because of the high temperatures at the SMA strip. The complete concrete beam, the strengthening strips and the clamping system were painted white with black speckles to achieve a high contrast, high quality speckle patterns for the digital image correlation (DIC) analysis.

## 3. Results

### 3.1. Results of the Activating Procedure

The SMA strip was heated and cooled as explained above. Due to the fact, that the strip was fixed to the concrete beam the reshaping caused by the SME was partly restricted. The SME creates a recovery stress in the SMA strip, which works as external prestressing of the tension zone of the RC beam in bending.

**Figure 8:**
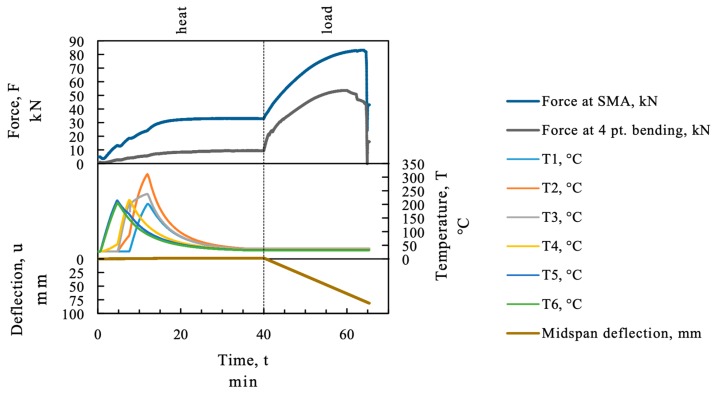
Temperature and recovery force at the SMA strip; beam SMA_1.

As mentioned before, the SMA expands in the first phase of heating. The thermal expansion before *A_s_* can lead to geometrical incompatibilities between the SMA and the corresponding existing structure if the whole strip is heated at once. The sequential heating procedure using an infrared emitter avoids a homogenous temperature profile throughout the SMA strip as can be seen in Figure 8 for the beam “SMA_1”. The maximum loss in force caused by thermal elongation of the strip in the first heating sequence was below 1 kN (about 8.5 MPa). In the continuing sequences, the force drop due to thermal expansion was even less because it was partially compensated by the cooling process of the previously activated SMA sequences.

Figure 8 displays the measured data, force, temperature, and midspan deflection over time. The recovery stress in the SMA was measured indirectly as a force with a load cell as recovery force. The force of the SMA is displayed in the upper section of Figure 8 on the force axes together with the force in the four point bending test setup. In the activation process the heating was stopped manually when the temperature measurement of the thermocouple reached about 200 °C. As can be seen in the temperature section in Figure 8 the SMA strip was heated to a higher temperature at thermocouple “T2”. At position “T2” the temperature started to rise from minute 5 (start of second heating sequence) due to heat transfer in the strip. In the third heating step the area of thermocouple “T2” was heated again by the emitter until thermocouple “T1” reached 200 °C and therefore overheated.

An increase of the load at the loading points of the four point bending test can be measured, because the displacement in the four point bending test frame was restricted during activation. The displacement in the test is plotted in in the bottom section of Figure 8.

**Figure 9:**
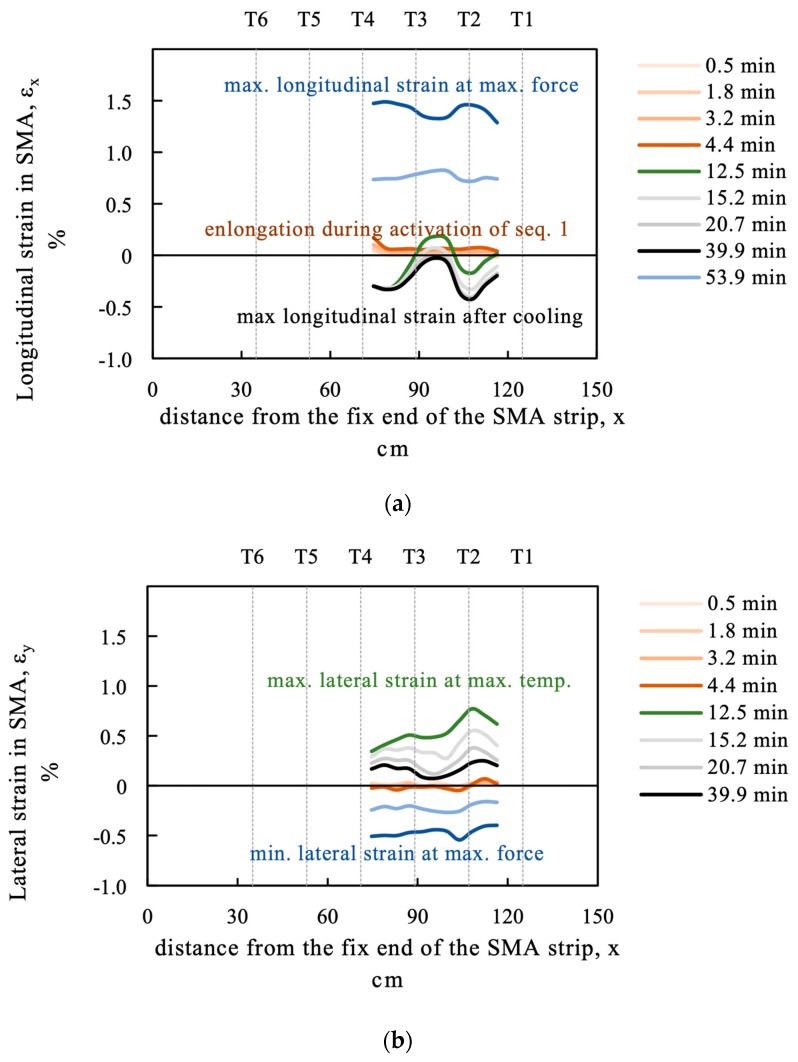
(**a**) Longitudinal and (**b**) lateral strain measurement at the SMA strip obtained from digital image correlation (DIC) at beam SMA_1.

Figure 9 shows a strain measurement at the SMA strip. Some of the variations in the obtained values might be due to different thermal treatment, but also varying cross-sections and material properties might influence the result. This measurement was obtained from DIC and therefore no data is recorded when the heater covers the SMA strip. The position of the strain measurement can be seen in Figure 14.

**Figure 10:**
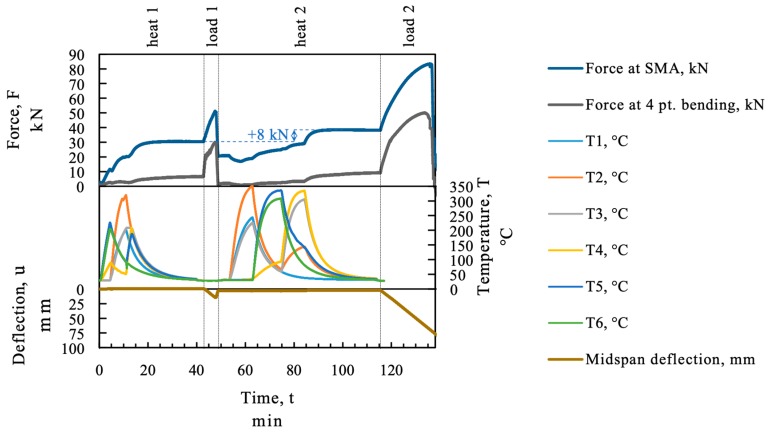
Temperature and recovery force at the SMA strip SMA_2.

Figure 10 shows the test procedure for beam “SMA_2”. The SMA mounted on this beam was activated by heating to at least 200 °C, the beam was loaded to 30 kN and unloaded. Afterwards the SMA was activated again at higher temperature (300–350 °C) and the beam was loaded to failure. The second activation generated a higher recovery force (+8 kN) therefore more of the SMA might have been transformed to austenite than in the first activation.

### 3.2. Results of the Four Point Bending Test

**Figure 11:**
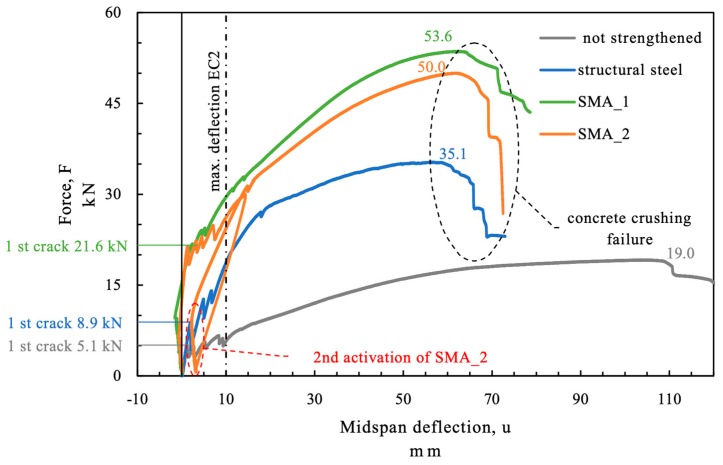
Load displacement behavior of the (strengthened) RC beams in the test.

**Figure 12:**
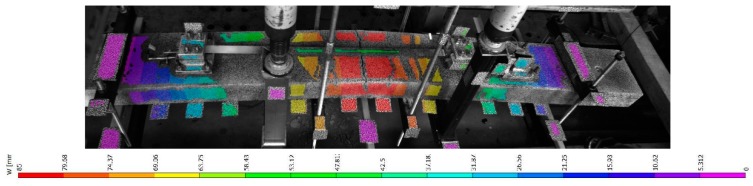
Vertical displacement of beam SMA_1 obtained from DIC.

During the four point bending test the force at the beam and the vertical displacement was measured. The force displacement curves of the beams are displayed in Figure 11. The corresponding vertical deformation field as obtained by DIC of beam “SMA_1 is given in Figure 12.

The force at the beam without additional strengthening (beam “not strengthened”) was 5.1 kN when the first cracks in the concrete occurred and 19 kN at ultimate load. The crack load for the beam strengthened with structural steel (beam “structural steel”) was 8.9 kN (+75%) and the ultimate load 35.1 kN (+85%). The first crack in the SMA strengthened beams (beams “SMA_1” and “SMA_2”) occurred at much higher load (over 21.5 kN +320%) due to the prestressing effect of the activated SMA. The failure loads of the SMA strengthened beams were above 50 kN (+160%). The maximum deflection limit in the serviceability limit state according to Eurocode is given by the effective length divided by 250, see EN 1990 [21] and EN 1992 [22]. For a span of 2.5 m this results in a maximum deflection of 10 mm. The maximum force at the deflection limit was highest for the SMA strengthened beams. At the beam “SMA_2” the alloy was activated a second time after the concrete beam had been loaded once to 30 kN. The second activation of the SMA could reduce the midspan deflection of the beam under service load compared to an imaginary loading without reactivating the SMA.

What can also be seen in Figure 11 is that the prestressing caused a negative deflection of the beam although the test setup was restrained. This is due to free deformation of the beam between the four points and because of the limited stiffness of the test frame.

The values of the loads especially the difference in ultimate load between the SMA strengthened beams (beams “SMA_1” and “SMA_2”) is of course not statistically significant due to the small number of tests and the variation of uncontrolled variables.

## 4. Discussion

The sequential activation procedure of SMA causes a more complex behavior than simultaneous heating obtained by resistive heating. One issue with sequential heating is that the activation of the SMA is not done under equal thermal and mechanical boundary conditions in every sequence. During the first activation sequence the alloy produces a recovery stress in the heated region but the neighboring regions of the alloy which stay cold act as a springs and expand. Therefore, the SME in the heated region is partially generating recovery stress and partially recovery strain (see green dotted line in Figure 2a).

Since the stress in the SMA strip increases from sequence to sequence, the affordable temperatures to induce the phase change *A_s_* and to finish the phase change *A_f_* are increasing as can be seen from the dashed lines in Figure 2b. This effect is also described in [23]. For inhomogeneously heated SMA strips a higher activation temperature below *A_f_* may increase the total transformation from martensite to austenite as long as the temperature stress state of the material does not exceed the limit of irreversible plasticity which is indicated by the dotted line in Figure 2b.

The cross-section of the strip is also affected by the phase transformation. After activation the cross-section is larger in the activated sequence and smaller in the sections remaining cool, which act as springs (see Figure 9a). The smaller cross-section does also contribute to a higher stress level and therefore higher target temperatures for phase transformation. Figure 9b shows the lateral strain of the SMA strip.

After the target temperature in the first section is reached the emitter is moved to the second sequence. The force in the SMA strip is constant over the strip length but does still increase until the strip is cooling down. This implies a higher stress state during the heating of the second sequence. As can be seen from Figure 2b, the stress state in the material affects the transformation temperatures. At this higher stress level higher temperatures are needed to obtain the same level of phase transformation.

**Figure 13:**
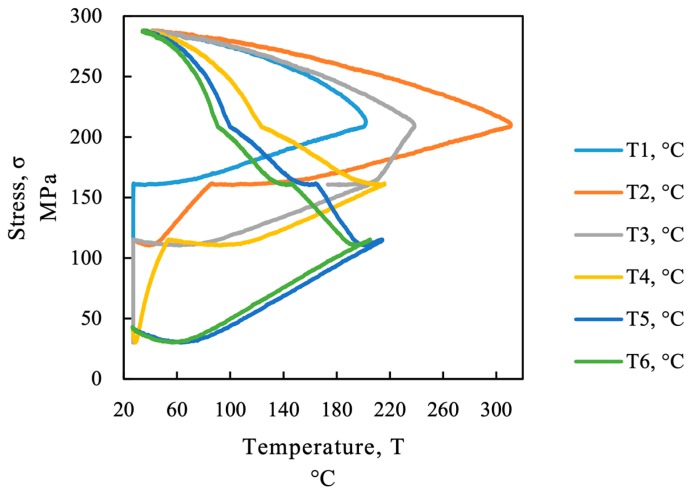
Temperature stress relationship at the SMA strip; beam SMA_1.

In Figure 13 the temperature and the stress in the SMA are plotted for the temperature measurement points of beam SMA_1. The stress is calculated neglecting the above-mentioned change in cross-section, due to the fact, that the true cross-sections could not be monitored during the whole heating and cooling process in every cross-section. As can be seen from Figure 13 the thermocouples T5 and T6 are heated in the first sequence and reach a maximum temperature of approximately 220 °C at about 100 MPa. The other thermocouples reach their maximum temperature at a much higher stress level. With respect to the theory of temperature and stress-dependent phase transformation, this indicates a different longitudinal activation level. The overheating at T2 which was described above, can also be seen in Figure 13. With respect to the stress- and temperature-dependent phase transformation the high temperature at T2 might not be as crucial as the low temperature at T1. This matter is discussed in Section 5 where temperature and phase transformation is modeled.

## 5. Modeling Activation Temperature and SMA Phase State

**Figure 14:**
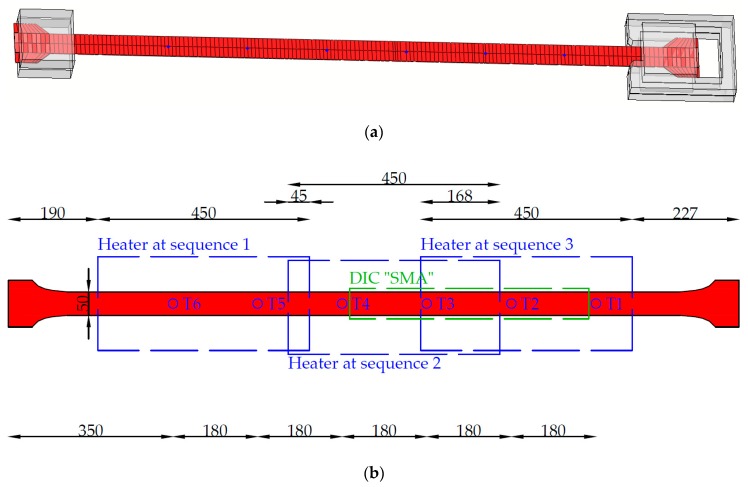
Thermal finite element (FE)-model, (**a**) mesh, and (**b**) geometry and monitoring points, sample SMA_1.

In order to determine the effect of stress and temperature state in every material point on phase transformation a finite element (FE) model is set up and calibrated on the measured temperatures for beam “SMA_1”. The FE calculations are performed with the commercially available software ABAQUS 2017 [24]. The SMA strip is modeled together with the metal clamps in order to capture the energy loss at the clamping device due to its high thermal conductivity. Eight-node linear brick elements DC3D8 are used for the steel and the SMA parts. A general mesh size of 40 mm is used and a mesh refinement of 10 mm along the SMA strip is made. The modeled geometry is shown in Figure 14.

The temperature in the test was measured at 6 single points with type K thermocouples. The thermal FE-model is used to calculate the temperatures between the measurement points. The thermal material properties of the steel and the SMA have not been tested. The following thermal material parameters have been set for the SMA: Thermal conductivity, *λ_SMA_* = 80 W/m/K; Density *ρ_SMA_* = 7860 kg/m^3^; Specific heat *c_SMA_* = 0.5 J/kg/K.

**Figure 15:**
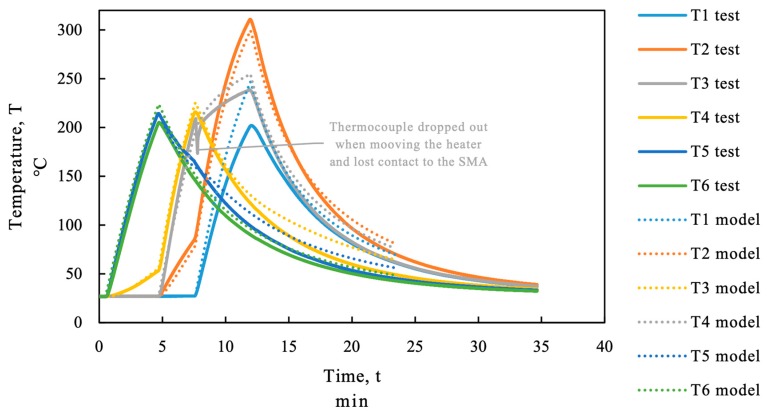
Comparison of the measured temperature and the modeled temperature; beam SMA_1.

The infrared heating is modeled as a surface radiation interaction with an ambient temperature of 360 °C in the first, 380 °C in the second and 400 °C in the third heating sequence. The emissivity during heating is considered to be 1 in all sequences. The thermal insulation below the thermocouple was modeled with reduced emissivity of 0.25. The cooling process was also modeled as a surface radiation interaction with an emissivity coefficient of 1 and ambient temperature of 27 °C. The modeled temperature (dotted line in Figure 15) at the monitoring points T1 to T6 is compared to the temperature measured in the test (continuous line in Figure 15). The low aberrations especially in the third heating section (T1–T3) and during the cooling process are accepted because the exact position and temperature of the heating device were not known and the ambient temperature in the cooling process was not controlled.

**Figure 16:**
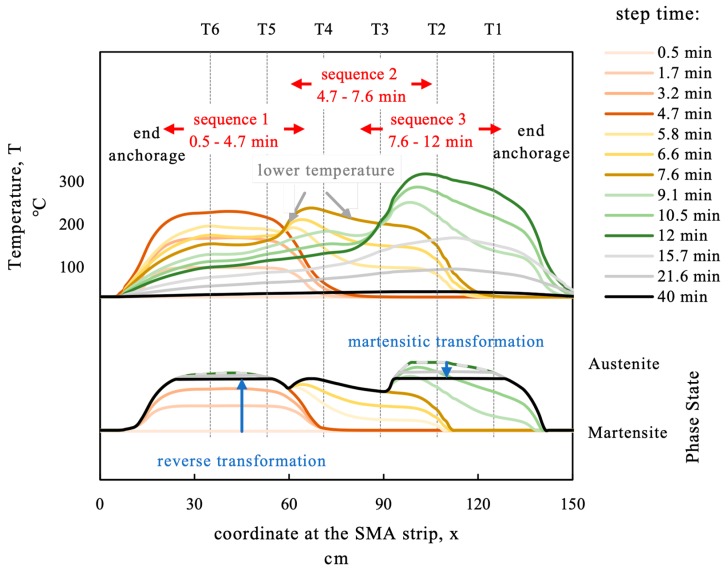
Comparison of the measured temperature and the modeled temperature.

The FE-model allows to calculate the temperature between the measured points. The modeled longitudinal temperature profile at the SMA strip is displayed for selected time steps in the upper section of Figure 16. It can be seen in the FE-model that the end anchorage was not heated. The heating process from 0.5 to 12 min can be seen for the three heating sequences. Except from the cold ends at the clamping and anchorage device the maximum temperature varies widely from about 200 °C (in the overlapping areas in between the heating sections) up to more than 300 °C in the third heating sequence. During cooling (from 12 to 40 min in Figure 16) the temperature profile is getting more and more homogenous due to thermal conductivity and the higher radiation rate at the higher temperature gradient.

As explained in Figure 2b phase transformation in the theory of SMA can be defined as a function of temperature and stress. With the temperature profile and the stress state in the strip a phase state can be calculated if the phase transformation temperatures are known. The stress state was calculated from the measured force and the nominal cross section neglecting the varying cross-section due to thermal expansion. For the used batch of Fe-SMA the phase transformation temperatures have not been tested. The calculation is based on the following functions for the phase transformation temperatures:

*A_s_* = 0.5 °C/MPa × σ + 5 °C; *A_f_* = 0.5 °C/MPa × σ + 200 °C; *M_s_* = 2.5 °C/MPa × σ − 525 °C; *M_f_* = 2.5 °C/MPa × σ − 1180 °C.

The values for *A_s_*, *A_f_*, and *M_s_* are chosen in compliance with the test results in [17]. Knowing that these values might vary for the material used in this study, the axis for phase state in Figure 16 is qualitative only. From minute 0.5 to minute 4.7 heating in sequence 1 takes place, as can be seen in the upper part of Figure 16.

The reverse transformation (martensite to austenite) in Section 1 can be seen in the lower part of Figure 16. The reverse transformation process (martensite to austenite) ends with removing the heater from sequence 3 at minute 12. During the cooling process similar to regular metals thermal contraction occurs in an SMA. Since the SMA strip is fixed at the ends, this contraction is inhibited and the stress increases. If the stress temperature state reaches the martensite start criterion a transformation from austenite to martensite occurs. This is depicted as martensitic transformation in Figure 2b. This transformation happens at the sections with the highest austenite content if the temperature in the strip is homogenous. The modeled phase transformation curve for minute 40 (black line in Figure 16) shows that the phase state profile levels itself in the previously overheated section. The magnitude of martensitic transformation depends on the considered phase transformation functions for the martensitic transformation.

## 6. Conclusions and Outlook

In this research two RC-beams were strengthened with iron based shape memory alloys which are prestressed by infrared heating and tested in four point bending tests. Two reference beams are tested. One strengthened with structural steel and one without any strengthening.

The strengthening with SMA strips increases the ultimate load by up to +160% and the load at first cracking could be increased up to +320%. Also the load at the serviceability limit could be enhanced clearly compared to the not strengthened and the structural steel strengthened beams, due to the prestressing effect of the SMA.

One beam was loaded in two cycles whereby the SMA was activated before each loading. The first loading cycle was intended to simulate a pre-existing damage including remaining deformation of the SMA. This deformation was undone by a second activation of the SMA. Due to the higher activation temperatures the prestressing was even higher than in the first loading cycle. The bending stiffness of the beam could be recovered partly by this prestressing. Therefore, the second activation of the pre-damaged beam was able to reduce the deformations under serviceability conditions.

The effect of different stress and temperature states and therefore different phase transformation temperatures along the longitudinal section is monitored and discussed in this publication. The effect of sequential activation influences the prestressing level but it obviously does not influence the failure mode since at high stress levels in the SMA strip the phase is transformed to martensite again. The activation of the SMA using an infrared emitter appeared to be a practical solution for external mounted SMA strips.

Since prestressing is dependent on stress and activation temperature a system for temperature and/or stress monitoring should be developed, to control the quality of application on site.

As mentioned before, the sequential activation caused a wide variation in temperature in the activated SMA. It could be shown that the different activation temperatures and stress conditions in the SMA did not lead to failure of the SMA. The varying temperature might not be a problem as long as the material is not damaged by the temperature stress state (e.g., irreversible plastic slip).

The anchorage and clamping device used in this study was designed for this special application with monitoring requirements in mind and is not intended to be a practical solution for strengthening applications.

The material and system behavior under temperature influence, sustained loads (relaxation) and fatigue still has to be verified for this alloy. Further research is needed on the varying phase state caused by the sequential activation including the effect on relaxation and fatigue behavior.

## Figures and Tables

**Table 1:** Concrete composition used for the reinforced concrete (RC) beam.

Component	Type	kg/m^3^
Cement	CEM II/A-LL 42.5 R	385
Gravel	8/16	675
Gravel	2/8	455
Sand	0/2	730
Water	-	168
Superplasticizer	BASF SKY 643	1.93

**Table 2:** Aggregate classification used in the concrete.

Component	Surface	wt.%
Carbonat	Smooth	27.5
Quartz and quartzite	Smooth	24.3
Magmatic	Smooth	16.2
Gneiss and other metamorphic components	Smoothrough	3.8
Sandstone greywacke	Smooth–rough	23.8
Hornstone radiolarite and silicate slate	Smooth	4.0
Other	-	0.4

**Table 3:** Tested concrete material parameters.

Material Parameter	Mean Value	Coefficient of Variation
Concrete compressive strength	58 MPa	3.5%
Concrete tensile strength	6.5 MPa	1.6%
Fracture energy	196 MPa	4.3%

**Table 4:** Residuals strain in the SMA after prestraining and unloading.

SMA-Strip Used in Beam No.	Residual Strain after Unloading ^1^	Unit
Structural steel 500	- ^2^	mm/m
SMA_1	36	mm/m
SMA_2	32	mm/m

^1^ The residual strain after unloading consists of plastic strain and recovery strain as stated in [15]. ^2^ Structural steel is used for strengthening, therefore the strip is not pre-strained.

**Table 5:** Test procedure for the RC beams in four point bending.

Test	Strengthening	Testing Procedure
not strengthened	no strengthening	loading the beam up to ultimate load
structural steel	structural steel *f_y_* ≅ 500	loading the beam up to ultimate load
SMA_1	SMA strip	1. activating SMA ~200 °C (restricted beam deflection)
		2. loading the beam up to ultimate load
SMA_2	SMA strip	1. activating SMA ~200 °C (restricted beam deflection)
		2. loading beam to 30 kN
		3. unloading of the beam
		4. reactivating SMA ~350 °C (restricted beam deflection)
		5. loading the beam up to ultimate load
